# Neural network-based clustering model of ischemic stroke patients with a maximally distinct distribution of 1-year vascular outcomes

**DOI:** 10.1038/s41598-022-13636-w

**Published:** 2022-06-08

**Authors:** Joon-Tae Kim, Nu Ri Kim, Su Hoon Choi, Seungwon Oh, Man-Seok Park, Seung-Han Lee, Byeong C. Kim, Jonghyun Choi, Min Soo Kim

**Affiliations:** 1 Department of Neurology, Gwangju-Jeonnam Regional Cerebrovascular Center, Chonnam National University Medical School, Chonnam National University Hospital, 42 Jebongro, Dong-gu, Gwangju, 61469 Korea; 2 Department of Mathematics and Statistics, Chonnam National University, 77 Yongbongro, Buk-gu, Gwangju, 61186 Korea; 3 AI Graduate School, Gwangju Institute of Science and Technology, Gwangju, Korea

**Keywords:** Stroke, Neurological disorders

## Abstract

Clustering stroke patients with similar characteristics to predict subsequent vascular outcome events is critical. This study aimed to compare several clustering methods, particularly a deep neural network-based model, and identify the best clustering method with a maximally distinct 1-year outcome in patients with ischemic stroke. Prospective stroke registry data from a comprehensive stroke center from January 2011 to July 2018 were retrospectively analyzed. Patients with acute ischemic stroke within 7 days of onset were included. The primary outcomes were the composite of all strokes (either hemorrhagic or ischemic), myocardial infarction, and all-cause mortality within one year. Neural network-based clustering models (deep lifetime clustering) were compared with other clustering models (k-prototype and semi-supervised clustering, SSC) and a conventional risk score (Stroke Prognostic Instrument-II, SPI-II) to obtain a distinct distribution of 1-year vascular events. Ultimately, 7,650 patients were included, and the 1-year primary outcome event occurred in 13.1%. The DLC-Kuiper UB model had a significantly higher C-index (0.674), log-rank score (153.1), and Brier score (0.08) than the other cluster models (SSC and DLC-MMD) and the SPI-II score. There were significant differences in primary outcome events among the 3 clusters (41.7%, 13.4%, and 6.5% in clusters 0, 1, and 2, respectively) when the DLC-Kuiper UB model was used. A neural network-based clustering model, the DLC-Kuiper UB model, can improve the clustering of stroke patients with a maximally distinct distribution of 1-year vascular outcomes among each cluster. Further studies are warranted to validate this deep neural network-based clustering model in ischemic stroke.

## Introduction

Stroke is one of the leading causes of mortality and disability in elderly patients^1,2^. The risk of recurrent vascular events is substantially high within the first several months after an index stroke^3^. Therefore, research on the early prediction of recurrent stroke and identification of high-risk patients is of great importance. Several markers, including clinical, radiological and laboratory findings^4^ and scoring methods, such as Stroke Prognostic Instruments (SPI)-II or the Essen Stroke Risk Score (ESRS), have been reported to be associated with recurrent events^5–7^. SPI-II and ESRS are commonly used and reliable risk scores because they are relatively simple and intuitively expressed through clinically important variables. However, the applications of conventional risk scores were limited because complex interactions among diverse factors, such as large artery steno-occlusion, stroke subtypes, and initial stroke severity, might exist in real clinical settings. If more variables are used, the degree of risk can be distinguished more effectively, but it will be more complex than conventional indicators. Artificial intelligence might provide a technical solution to these difficulties.

In recent years, AI techniques have been used in an increasing number of stroke-related studies. The main areas of AI stroke studies are outcome prediction, diagnosis and treatments^8,9^. The identification of similar clusters according to “time-to-event” also plays an important role in stroke care and future research. However, AI clustering models remain a relatively unexplored topic in stroke fields. Clinically, large datasets comprise phenotypically heterogeneous subpopulations—i.e., subsets of observations that cluster according to both covariates and time-to-event similarities. For example, in a clinical setting, identifying high-, medium-, and low-risk subpopulations equipped with accurate time-to-event estimates can allow more appropriate targeting of interventions, treatments or care delivery. Therefore the use of neural network-based models to identify heterogeneous subpopulations in acute ischemic stroke with different time-to-event risks (survival) would be a crucial step toward precision medicine^10,11^.

Several lifetime clustering methods exist, such as traditional unsupervised clustering methods, including k-means^12^, semi-supervised clustering^13^, and supervised sparse clustering methods^14^. Recently, a neural network-based lifetime clustering model, *DeepCLife*, was introduced^15^ that could identify cluster assignments by directly maximizing the divergence between the empirical lifetime distributions of the clusters.

This study attempted to show a clearer prognostic difference using more information and more variables than those used for SPI-II scores. Thus, a clustering method was used to create risk groups and compare them. Additionally, we compared several lifetime clustering methods, including the deep lifetime clustering (DLC) method, and assessed the performance of the clustering methods on a real-world survival dataset of patients with ischemic stroke.

## Methods

### Study subjects

This study analyzed data from a single regional comprehensive stroke center registry of consecutive patients with acute ischemic stroke. This registry is part of the Clinical Research Collaboration for Stroke-Korea (CRCS-K) registry^16,17^. A total of 8,136 patients with ischemic symptoms who were admitted to our tertiary stroke center between January 2011 and July 2018 were initially screened. We included patients with ischemic stroke within 7 days of onset. The detailed eligibility criteria and patient selection flow chart are shown in Supplemental Fig. 1.

**Figure 1:**
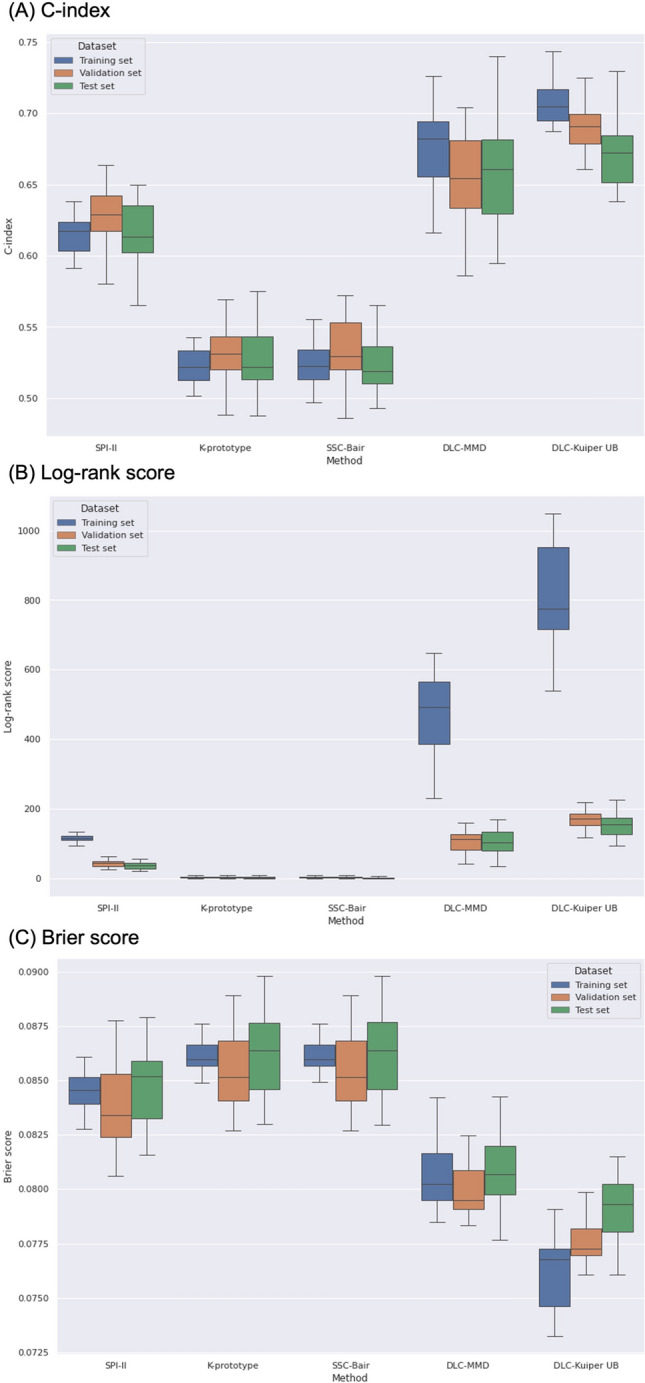
C-index (**A**), log-rank score (**B**), and Brier score (**C**) for different clustering methods applied to the datasets.

### Ethics statement

Clinical information was obtained from the registry database. The registry has been collecting clinical information for the purpose of monitoring and improving the quality of stroke care since 2011, with the approval of the institutional review boards of our hospital. The use of the registry database and the supplemental review of medical records in this study were approved by the institutional review boards. A waiver for informed consent was provided because of study subject anonymity and minimal risk to the participants by an institutional review board at Chonnam National University Hospital. All the methods were performed in accordance with the relevant guidelines and regulations (Declaration of Helsinki). The corresponding author will provide the data, analytical methods, and study materials to other researchers upon reasonable request. Additionally, the GitHub website address for the DLC method is as follows: (https://github.com/PurdueMINDS/DeepLifetimeClustering).

### Data collection

Data were prospectively collected and included demographic, clinical, imaging and laboratory data. Data collection is described in the Supplemental Methods. For continuous variables, if fewer than 3% of the values were missing, the data were imputed with the median values or as the mode.

### Outcomes

The primary outcome was a composite of all types of recurrent stroke (ischemic and hemorrhagic), myocardial infarction, and all-cause mortality within one year after the index stroke. Secondary outcomes consisted of the following individual events: (1) all types of recurrent stroke (ischemic and hemorrhagic) and (2) all-cause mortality. As previously described^17^, vascular events were prospectively captured during hospitalization and the 3-month and 1-year follow-up periods by dedicated stroke nurses or physicians based on predefined protocols at routine clinic visits or during telephone interviews. To ensure the accuracy of the outcomes and to minimize inter-interviewer discrepancies, a uniformly structured questionnaire was used by trained personnel^17^.

### Clustering models

The clustering method is a type of unsupervised learning that consists of similar characteristics within a group and different characteristics between groups through the characteristics of individuals. This means that there are no actual labels, but clustering gives each object a new label (a risk-based cluster in the study). The criteria for dividing the degree of risk for the generated clusters are the Kaplan–Meier curve.

As a reference in stroke patient clustering, the SPI-II scores were used. The SPI-II scores were retrospectively calculated as the sum score (0–15 points) of 7 clinical factors based on their predictive significance: congestive heart failure (3 points), diabetes mellitus (3 points), prior stroke (3 points), age > 70 years (2 points), stroke as the index event (2 points), severe hypertension (1 point), and coronary artery disease (1 point)^5^. To investigate the risk of 1-year vascular events, the SPI-II scores were categorized into 3 groups based on previous studies: low risk (0–3), medium risk (4–7), and high risk (> 8).

#### K-prototype

K-means clustering is a representative clustering method that assigns a given datapoint to preset k clusters. The cluster is updated until no further cluster changes occur in the direction of minimizing the sum of squares of the center of each cluster and the distance between objects. However, K-means clustering can be used in continuous data. The method used for categorical data is called k-mode clustering, which replaces the means of clusters with modes. K-prototypes is a clustering method that combines K-means and K-modes methods for use in a mixture of continuous and categorical data^12^.

#### SSC-Bair

SSC-Bair is a clustering model that groups using K-means by selecting covariates that affect the hazard ratio through Cox models^13^.

#### DLC model

DLC proceeds with survival clustering to utilize neural networks to optimize differences between empirical lifetimes. Neural networks are learned through covariates of observations, and the final layer of neural networks is allocated to one of the k-clusters through softmax layers. The empirical lifetime distribution is obtained from the Kaplan–Meier estimator using the assigned clusters, and the model is trained to maximize the difference in the survival distribution of the clusters. Because DLC does not assume a proportional risk, it can cross a lifetime curve. The objective function of the DLC model is as follows:

$$W_{1}^{*} , W_{2}^{*} = \mathop {{\text{argmax}}}\limits_{{W_{1} ,W_{2} }} \mathop {\min }\limits_{{i,j \in \left\{ {1 \ldots K} \right\}, i \ne j}} {\Delta }\left( {\hat{S}_{i} ,\hat{S}_{j} } \right)$$

$\hat{S}$ is an empirical distribution, and ∆ is the divergence measure. However, because datasets have information concerning the occurrence of events in this study, $W_{2}$, indicates that a stochastic estimation of the terminating signal is not considered^15^.

Although many methods are available to measure divergence, sample sizes must be considered. The gradient calculation should be simple because it serves as an objective function of neural networks. Additionally, proportional hazards should not be assumed. We used and compared the Kuiper *p*-value upper bound (Kuiper UB) and maximum mean discrepancy (MMD) as divergence measures^18,19^.

### Shapley Additive exPlanations (SHAP)

We applied the Shapley Additive ExPlanations (SHAP) method to verify the importance of variables for each cluster. SHAP is a unified framework for predictive interpretation^20^, which expresses the importance of variables by comparing predictions of situations with baseline values when there are certain values for a given feature.

### Detailed instruction of applied models

The percentage of missing values for variables was less than 3%, the categorical variables were replaced by values that did not result in events, and the continuous variables were replaced by median values for nonmissing data. The analysis was conducted after preprocessing each method. Among the application methods, SPI-II scores distinguished each group by labeling divided by scores. For comparison with groups of SPI-II scores, the number of groups in other models was set to 3. SSC-Bair selected only variables with *p*-values less than 0.1 through the Cox model, classifying them into three clusters through k-means clustering. DLC is a neural network-based clustering model that used 30 characteristics in this study (Table 1). DLC included the patient's gender and age as input, and information on whether an event occurs within a year of the patient and the time of event occurrence as a target. Continuous input variables were scaled using a minimax scaler, and categorical variables were analyzed as one-hot encoding. By optimizing the loss function that maximizes the difference in the empirical distribution of K (set to 3 in this study) clusters through input and target, weights are updated to allocate patients to three clusters considering the characteristics and lifetime distribution. Because the results differed according to the change in the hyperparameters, the final model was selected by the validation set after the experiment by changing the hyperparameters such as the number of layers, learning rate, and activation function (Supplemental Table 1). Using the final selection model, when a new patient occurs, the risk group of the patient can be presented by entering the patient's characteristics. SHAP used DeepExplainer to perform the analysis among the Explorers provided by the library. In general, a dataset is divided into training, validation, and test sets. The training set is required for learning to generate a model, and the validation set is used to avoid overfitting of the learned model and determine appropriate hyperparameters. The pre-set hyperparameters (such as those shown in Supplementary Table 1 for the DLC model) are applied to the model learned in each combination. From these many candidate models, the model with the best performance based on the evaluation index is selected and used as the final model. The test set is used to evaluate the performance of the final model selected using the validation set. The training, validation, and test sets were divided 60:20:20 using stratified sampling based on the outcome rate to reduce bias.

**Table 1:** General characteristics of subjects.

Variables	Values
N	7650
Age, yr, mean (SD)	68.6 (12.4)
Male, N (%)	4433 (57.9)
**Arrival time, N (%)**	
Within 24 h	5830 (76.2)
Beyond 24 h	1820 (23.8)
BMI, mean (SD)	23.5 (3.3)
Initial NIHSS, med (IQR)	3 (1, 9)
Prestroke disability (pre-mRS > 1), N (%)	1154 (15.1)
**TOAST classification, N (%)**	
LAA	2269 (29.7)
SVO	801 (10.5)
CE	1899 (24.8)
OE	125 (1.6)
UD	2556 (33.4)
**Medical history, N (%)**	
History of TIA	114 (1.5)
History of stroke	1308 (17.1)
History of peripheral artery diseases	47 (0.6)
History of coronary artery diseases	432 (5.6)
HTN	4435 (58.0)
DM	2078 (27.2)
Dyslipidemia	1190 (15.6)
Smoking	
Never	5128 (67.0)
Current	1496 (19.6)
Ex-smoker (quit > = 5 yr)	444 (5.8)
Recent smoker (quit < 5 yr)	582 (7.6)
Atrial fibrillation	1889 (24.7)
High risk of cardioembolism	1709 (22.3)
Congestive heart failure	31 (0.4)
**Medication history, N (%)**	
Antiplatelet	1750 (22.9)
Anticoagulant	337 (4.4)
Antihypertensive	3619 (47.3)
Antidiabetic	1696 (22.2)
Statin	855 (11.2)
**Laboratory findings, mean (SD)**	
White blood cell counts, 10^3^/µL	8.43 (3.13)
Hemoglobin, mg/dL	13.6 (1.9)
Platelet counts, 10^3^/µL	221.6 (65.9)
Glucose, mg/dL	136.6 (55.2)
Creatinine, mg/dL	0.90 (0.73)
Systolic blood pressure, mmHg	139.1 (114.0)
**Large artery disease, N (%)**	
No stenosis	3073 (40.2)
Mild stenosis < 50%	479 (6.3)
Moderate-to-severe stenosis > 50%	1171 (15.3)
Complete occlusion	2927 (38.3)

BMI; body mass index, NIHSS; National Institutes of Health Stroke Scale, TOAST; Trials of Org.

### Statistical methods

The data are reported as percentages, means (standard deviations), or medians (IQRs), depending on the variable characteristics. Categorical variables were analyzed using Pearson’s chi-squared test or Fisher’s exact test, and continuous variables were analyzed using analysis of variance or the Kruskal–Wallis test, as appropriate. The following parameters had missing data that were substituted using median values: BMI (2.2%), creatinine (0.2%), hemoglobin (0.1%), white blood cell count (0.1%), and initial random glucose (0.7%). The metrics used to evaluate the clusters obtained from the methods were the concordance index, Brier score, and Log-rank score test. The results of the metrics represented the mean and standard deviation in 30 iterations. In addition, we also conducted conventional statistical analyses for associations of each cluster with 1-year vascular outcomes according to the cluster methods in the study cohort. A *p*-value < 0.05 was considered statistically significant. All analyses were conducted in Python (version 3.7.9) and PyTorch (version 1.6.0).

### Ethics approval

The current study was approved by the institutional review board at Chonnam National University Hospital.

### Consent to participate

A waiver for informed consent was provided because of study subject anonymity and minimal risk to the participants by the institutional review board at Chonnam National University Hospital.

## Results

### General characteristics

Among 8136 patients with stroke registered in the registry during the study period, 7650 patients (age 68.6 ± 12.4 years, male 57.9%) were ultimately analyzed for the study (Supplemental Fig. 1). The median National Institutes of Health Stroke Scale (NIHSS) score was 3 (IQR 1–9) (Table 1). The proportions of the SPI-II risk groups were as follows: low risk (SPI-II score of 0–3), 30.3%; medium risk (SPI-II score of 4–7), 50.2%; and high risk (SPI-II scores > 7), 19.5%. In the DLC-Kuiper UB methods for the training sets, 3 distinct clusters (clusters 0, 1, and 2) were 10.3%, 44.4%, and 45.3%, respectively (Supplemental Table 2).

**Table 2:** C-index, Log-rank score, and Brier score for different clustering methods in datasets.

	SPI-II	K-prototype	SSC-Bair	DLC-MMD	DLC-Kuiper UB	*P*-value
**(A) Training set, mean (SD)**						
C-index	0.615 (0.015)	0.523 (0.012)	0.523 (0.013)	0.673 (0.030)	0.709 (0.019)	< 0.001
Log-rank score	116.990 (12.122)	4.061 (2.356)	4.515 (2.971)	476.833 (110.306)	817.736 (140.927)	< 0.001
Brier score	0.085 (0.001)	0.086 (0.001)	0.086 (0.001)	0.081 (0.002)	0.076 (0.002)	< 0.001
**(B) Validation set, mean (SD)**						
C-index	0.628 (0.022)	0.531 (0.018)	0.532 (0.024)	0.654 (0.032)	0.690 (0.018)	< 0.001
Log-rank score	43.690 (9.587)	3.555 (2.547)	3.673 (2.706)	106.983 (30.735)	170.533 (24.017)	< 0.001
Brier score	0.084 (0.002)	0.086 (0.002)	0.086 (0.002)	0.080 (0.001)	0.077 (0.001)	< 0.001
**(C) Test set, mean (SD)**						
C-index	0.614 (0.024)	0.525 (0.022)	0.524 (0.020)	0.657 (0.039)	0.674 (0.027)	< 0.001
Log-rank score	38.247 (9.315)	2.810 (2.769)	2.796 (2.753)	106.249 (33.097)	153.099 (32.740)	< 0.001
Brier score	0.085 (0.002)	0.086 (0.002)	0.086 (0.002)	0.081 (0.002)	0.079 (0.001)	< 0.001

*P*-value; One-Way ANOVA.

The performances of each clustering method are shown in Table 2 and Fig. 1. In the training, validation, and test sets, the DLC-Kuiper UB method produced significantly better clusters with distinct “time-to-composite vascular event” distributions, as evaluated by the C-index (0.709, 0.690, and 0.674, respectively), log-rank score (817.736, 170.533, and 153.099, respectively), and Brier score (0.076, 0.077, and 0.079, respectively), than the SPI-II, K-prototype, SSC-Bair, and DLC-MMD methods.

### Outcomes

The 1-year primary composite outcome events occurred in 1,005 (1-year cumulative event rate; 13.1%) patients. Regarding individual outcomes, the 1-year cumulative event rates were 2.9% for recurrent stroke and 10.7% for all-cause mortality. MI occurred in 0.2% of patients.

The one-year vascular events of each cluster according to the clustering methods are shown in Table 3 and Supplemental Tables 3 and 4. We set the rate of occurrence of events within the group as a criterion for dividing the risk groups. For the training, validation, and test sets of the DLC-Kuiper UB method, 3 clusters had significantly different 1-year primary vascular outcomes (training set: 53.7%, 10.7%, and 6.3%, validation set: 38.4%, 13.2%, and 7.2%, and test set: 41.7%, 13.4%, and 6.5%, respectively, all *P*-values < 0.001). As there are no actual labels of high-, medium-, and low risk in the unsupervised clustering method, the Kaplan–Meier curves for 3 clusters could generate risk groups based on the risk of 1-year vascular events. Therefore, Cluster 0 is judged as the high-risk group, Cluster 1 as the medium-risk group, and Cluster 2 as the low-risk group.

**Table 3:** One-year outcomes of patient groups according to the clustering method (k = 3/test set).

	Cluster 0	Cluster 1	Cluster 2	*P*-value
**(A) K-prototype**				
N	949	168	413	
Primary outcome	136 (14.3%)	16 (9.5%)	49 (11.9%)	0.158
Stroke	26 (2.7%)	3 (1.8%)	14 (3.4%)	0.557
MI	0 (0.0%)	0 (0.0%)	2 (0.5%)	0.067
All-cause mortality	117 (12.3%)	14 (8.3%)	37 (9.0%)	0.096
**(B) SSC-Bair**				
N	164	952	414	
Primary outcome	15 (9.1%)	136 (14.3%)	50 (12.1%)	0.150
Stroke	3 (1.8%)	26 (2.7%)	14 (3.4%)	0.579
MI	0 (0.0%)	0 (0.0%)	2 (0.5%)	0.067
All-cause mortality	13 (7.9%)	117 (12.3%)	38 (9.2%)	0.100
**(C) DLC-MMD**				
N	966	527	37	
Primary outcome	69 (7.1%)	117 (22.2%)	15 (40.5%)	< 0.001
Stroke	20 (2.1%)	23 (4.4%)	0 (0.0%)	0.022
MI	1 (0.1%)	1 (0.2%)	0 (0.0%)	0.885
All-cause mortality	52 (5.4%)	101 (19.2%)	15 (40.5%)	< 0.001
**(D) DLC-Kuiper**				
N	156	677	697	
Primary outcome	65 (41.7%)	91 (13.4%)	45 (6.5%)	< 0.001
Stroke	8 (5.1%)	23 (3.4%)	12 (1.7%)	0.031
MI	0 (0.0%)	2 (0.3%)	0 (0.0%)	0.283
All-cause mortality	63 (40.4%)	69 (10.2%)	36 (5.2%)	< 0.001

*P*-value; Chi-squared test.

**Figure 2:**
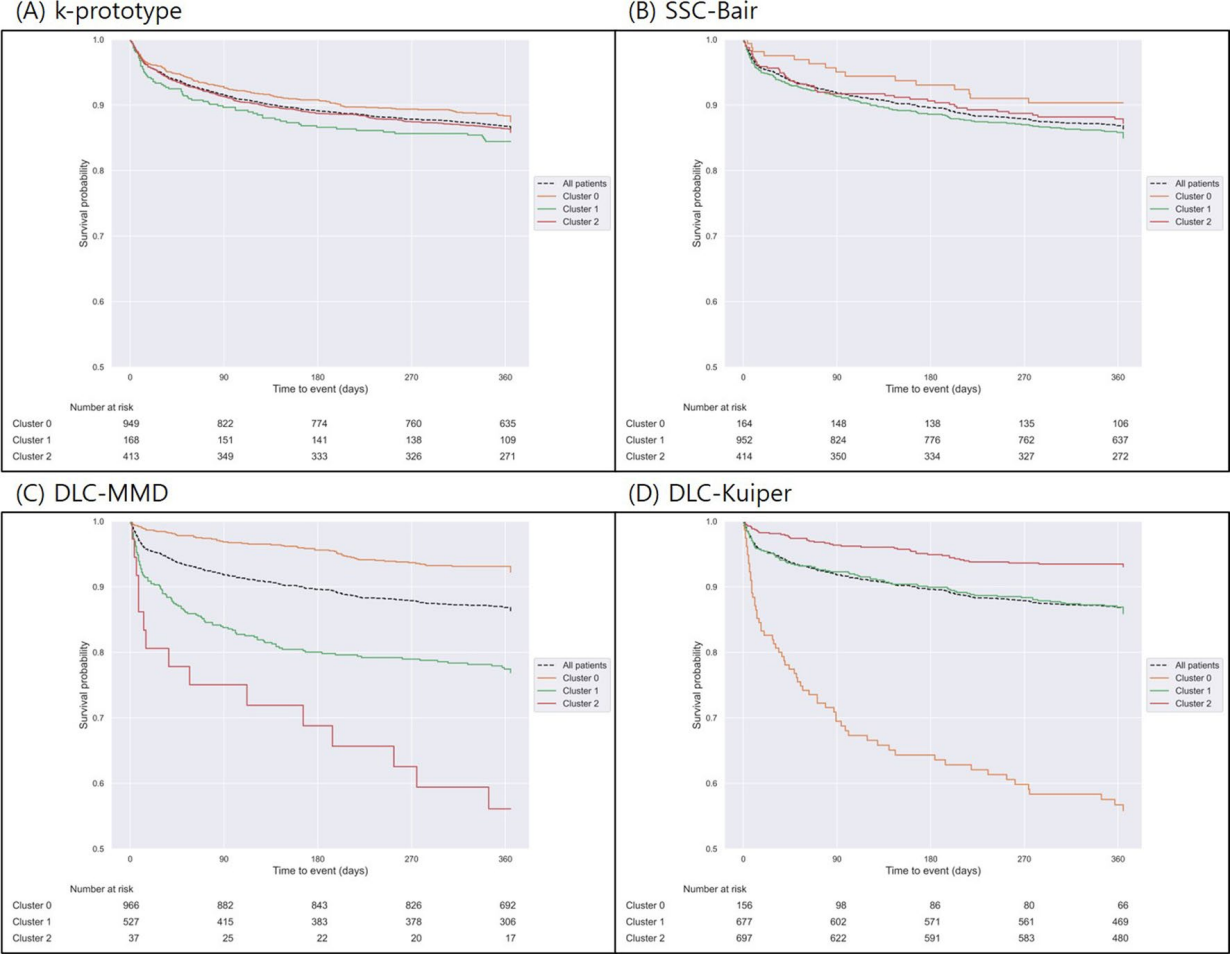
Kaplan–Meier curves of the primary outcome within 1 year from different clustering methods for K = 3 (3 clusters). (**A**) K-prototype, (**B**) SSC-Bair, (**C**) DLC-MMD, and (**D**) DLC-Kuiper UB, all test set results.

However, for the SSC-Bair method, the primary vascular outcome within 1 year was not significantly different among the 3 clusters. As a comparator, primary vascular outcomes within 1 year were significantly different among the SPI-II subgroups (5.6%, 14.7%, and 20.7% in the low-, medium-, and high-risk subgroups, respectively) (Supplemental Table 5). Kaplan–Meier survival curves according to the cluster methods are shown in Fig. 2.

### Identification of important factors

**Figure 3:**
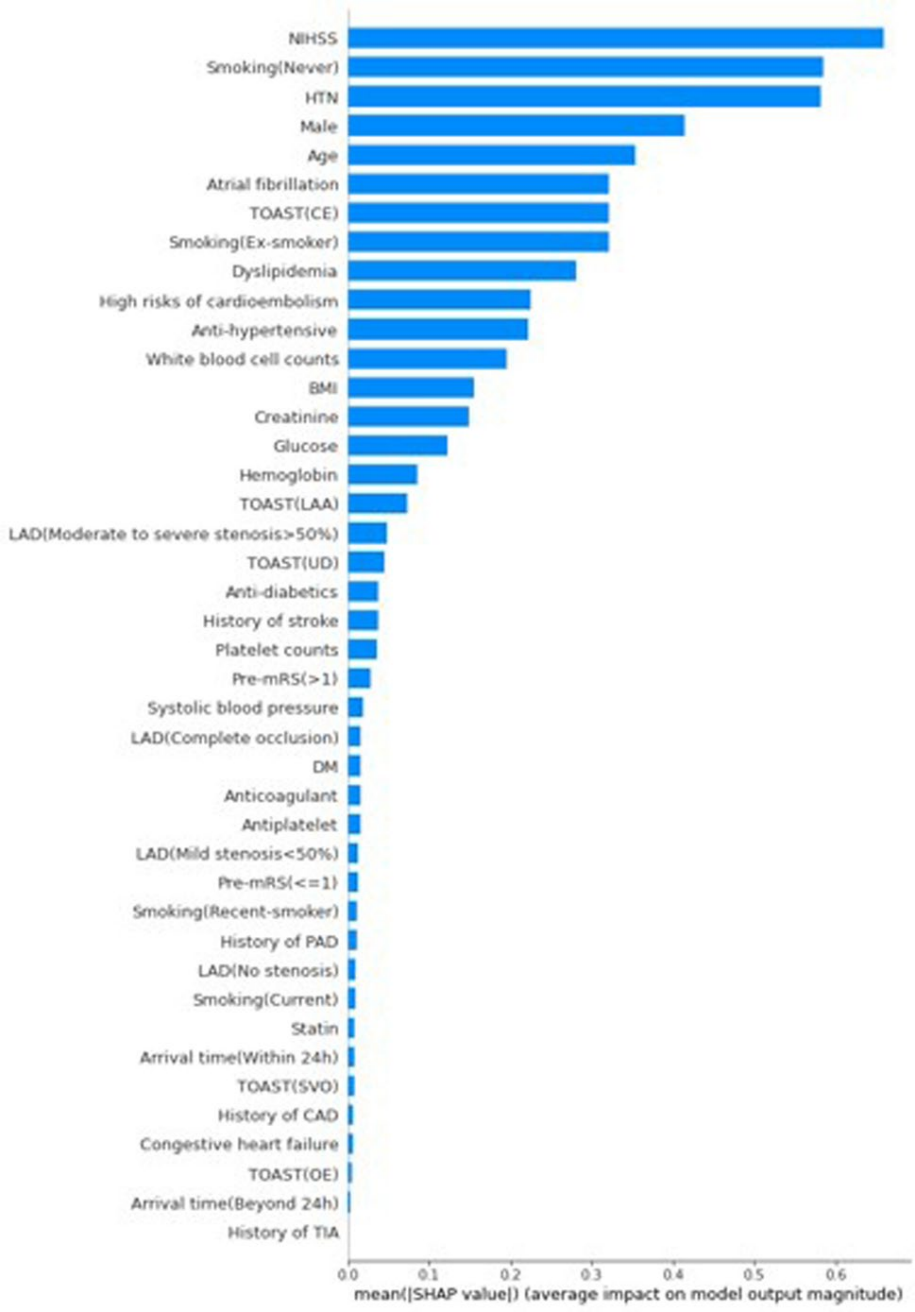
Feature importance matrix plots. In the bar plot, the SHAP value implies the degree of contribution of a specific feature. The higher the SHAP value is, the larger the model contribution of a specific feature.

SHAP feature importance matrix plots show the important features according to the degree of contribution (Fig. 3). Among them, initial NIHSS score, never smoking, and hypertension (HTN) contributed maximally to each cluster. Other features contributed to the model at a similar or decreasing level.

## Discussion

This study provides validation of the neural network-based clustering model, the DLC-Kuiper UB method, as a tool for stroke patient clustering with maximally distinct 1-year vascular outcome events. Compared with the SPI-II stroke risk score and other clustering methods, the DLC-Kuiper UB model presented significantly better clustering performance, with a high C-index, Log-rank score, and Brier score. These results suggest that the DLC-Kuiper UB method could be useful for grouping stroke patients with similar outcome risks. Further research with independent cohorts is warranted to confirm the results.

Finding similar clusters among stroke patients can be helpful from a medical perspective, as it may lead to the discovery of new patterns and more effective ways to manage stroke. Several studies have sought to identify clusters among stroke patients. Clustering stroke patients based on clinical data using the expectation–maximization algorithm to estimate the parameters of Gaussian mixture models resulted in two suitable clustering schemes that divided stroke patients into two and three clusters, respectively^21^. However, for the 3-months modified Rankin Scale score, significant variations were observed among the three clusters. Another study explored effective prediction models for discharge planning based on a machine learning algorithm derived from data gathered during hospitalization in the acute phase of ischemic stroke. That study found that the use of clustering learning algorithms permits the unsupervised identification of a set of clinical variables that achieve good prediction of clinical outcomes^22^.

However, clustering based on risk-profiles in survival analysis is relatively unexplored in machine learning but would be critical in applications such as (clinical) decision making. Several conventional risk scores for stratifying the risk of stroke recurrence exist^23,24^. The SPI-II is a validated and reliable stroke risk score^25^. However, classic tools to predict stroke outcome risks might have limitations because each possible variable cannot be considered. For example, the SPI-II could predict outcomes using only 7 clinical factors based on their predictive significance; congestive heart failure, diabetes mellitus, prior stroke, age > 70 years, stroke as the index event, severe hypertension, and coronary artery disease. However, there may be interactions with many crucial variables related to outcomes, such as large artery steno-occlusion, LAA or CE stroke subtypes, and initial stroke severity. Additionally, feature-based clustering in covariate space may produce clusters that are inconsistent with survival outcomes^13,26^, particularly high-dimensional datasets. Methods that account for survival outcomes in clustering include Cox Proportional Hazards (PH)-inspired techniques, but Cox PH-based approaches are limited by the proportional hazards assumptions.

Neural network-based clustering methods could automatically consider the significance/interaction of individual clinical factors. Xia et al. proposed an outcome-driven, attention-based multi-task deep learning model for classification and subtyping in patients with acute coronary syndrome^27^. With recent advances in machine learning, deep learning methods have improved classical survival analysis methods by leveraging non-linear relationship between covariates, for improved time-to-event or risk score predictions. Compared with classic methods such as the k-prototype and SSC-Bair methods, the DLC-Kuiper UB model is robust to the modeling issues associated with the inability to observe termination signals and does not assume proportional hazards^15^. Our results, therefore, found that the neural network-based DLC-Kuiper UB method could produce similar clusters with maximally distinctive 1-year vascular outcomes by using patient information on real-world stroke datasets. However, this method may be limited because it does not investigate clustering based on the severity of outcomes. Further validations for stroke subgroups, may also be warranted to confirm the results. In addition, the ability of the DLC-Kuiper UB model to incorporate more clinical variables to obtain better performance may not indicate a better AI-based DLC-Kuiper UB algorithm at the algorithmic level.

Better treatment has been determined by comparing the efficacy and safety of *A* treatment versus *B* treatment by randomized controlled trials. However, individually tailored treatment would eventually be needed in acute ischemic stroke due to diverse stroke mechanisms and different risks of recurrence. Before that is possible, identifying similar clusters in stroke patients would be helpful to determine which groups would be better in group-specific research, including treatment. Additionally, because ‘time-to-event’ would be unlabeled, clustering relies on unsupervised machine learning. If the examples are labeled such as stroke subtypes, then clustering becomes classification. Our study found that the DLC-Kuiper UB methods consistently clustered similar patients in both the training and test sets (C-index 0.709 and 0.674, respectively) compared with the classic methods. The results might be the basis for future research applying different treatments according to the different clusters.

As mentioned in the Introduction, survival clustering is important but is not being actively studied. Nevertheless, new methods continue to be proposed. Mouli et al. proposed a survival clustering method based on a decision tree prior to DeepCLife^15,28^. Liverani et al. proposed a Dirichlet process mixture model suitable for cases where it is difficult to apply usual survival models due to multicollinearity and identified clusters using both censored survival data and predictors^29^. Chapfuwa et al. proposed a time-to-event prediction model with structured latent representations that can be clustered through a prior for infinite mixture of distributions^30^. Among these various methods, our study used DLC because it was judged that a neural network-based model that maximizes the divergence of the empirical lifetime distribution among clusters considering censored events would more clearly distinguish groups according to the risk levels of stroke patients.

Our results additionally provide the level of contribution of specific input features selected from the model using the whole dataset as well as individual patient information by using SHAP. The features that contributed the most to clustering were the initial NIHSS score and age among all 3 clusters. Our results support previous studies, as NIHSS scores and age were very important factors in grouping stroke patients and predicting outcomes^31–33^. In the future, however, the addition of imaging findings, including brain MRI and clinical features, might improve the clustering and prediction of outcomes in acute ischemic stroke. In particular, further studies should be conducted only on patients with large vessel occlusions because these cases require endovascular treatment.

There are several limitations in our study. First, our study had the inherent limitation of including data from only a single stroke center registry in South Korea. Further studies using multicenter, multiethnic or multiracial datasets are warranted. Although datasets were divided and analyzed into training, validation, and test sets, further research with independent cohorts is warranted. In addition, the dataset was maintained at a constant outcome ratio through stratified sampling for analysis, but this may not necessarily be the case with a new dataset. Nonetheless, a neural network-based cluster model was first applied to stroke patients from a real-world dataset. Second, as available data in the registry might be limited, the results have limited generalizability. External validation would be warranted. Third, the clinical implications of clustering in stroke patients should be further investigated. Therefore, further verification and research are warranted by conducting well-designed clinical research in the future.

In conclusion, our study found that a neural network-based clustering method, the DLC-Kuiper UB model, could be a good tool for clustering stroke patients with similar 1-year vascular outcomes. Further studies are warranted to verify and validate this AI-based clustering model in ischemic stroke.

## Supplementary Information

Supplementary Information.

## Data Availability

The corresponding author will provide the data, analytic methods, and study materials to other researchers upon reasonable request.
