# Supplementary material for: Neural network-based clustering model of ischemic stroke patients with a maximally distinct distribution of 1-year vascular outcomes
Source: Sci Rep. 2022 Jun 8;12:9420. doi: 10.1038/s41598-022-13636-w (PMC9177616; doi:10.1038/s41598-022-13636-w)
Supplement: Supplementary file 1 — Supplementary Information. [file 41598_2022_13636_MOESM1_ESM.docx]

**Online Supplements**

Title: Neural network-based clustering model of ischemic stroke patients with a maximally distinct distribution of 1-year vascular outcomes

Authors: Joon-Tae Kim, MD, PhD^1^*, Nu Ri Kim, PhD,^1^* Su Hoon Choi, PhD,^2^* Seungwon Oh, PhD,^2^ Man-Seok Park, MD, PhD,^1^ Seung-Han Lee, MD, PhD,^1^ Byeong C. Kim, MD, PhD,^1^ Jonghyun Choi, PhD,^3^ Min Soo Kim, PhD ^2^

^1^Department of Neurology, Chonnam National University Hospital, Chonnam National University Medical School, Gwangju, Korea

^2^Department of Mathematics and Statistics, Chonnam National University, Gwangju, Korea

^3^AI Graduate School, Gwangju Institute of Science and Technology, Gwangju, Korea

* contributed equally as first authors

Subtitle: AI-based clustering in ischemic stroke

Supplemental Methods.

Supplemental Tables 1-5.

Supplemental Figure 1.

Supplemental Methods

*Data collection*

Data analyzed in this study were abstracted in the Clinical Research Center for Stroke-Korea (CRCS-K) registry, a prospective, multicenter, nationwide registry of consecutive patients with acute stroke or transient ischemic attack admitted to 16 stroke centers in South Korea. It is a web-based database.

The following data were obtained from the registry database: (1) demographics, including age, sex, and body mass index; (2) medical history, including previous TIA, previous stroke, previous coronary artery disease (CAD), previous peripheral artery disease (PAD), hypertension, diabetes mellitus, dyslipidemia, smoking (current, recent, and ex-smoker), atrial fibrillation, and potential embolic sources with high risk; (3) medications, including previous antiplatelet medications, previous antihypertensive medications, previous antidiabetic treatments, previous lipid-lowering medications other than statins, and previous statin medications; (4) stroke characteristics and acute treatments, including the onset-to-arrival time, initial NIHSS score, prestroke modified Rankin Scale (mRS) score, ischemic stroke subtype according to the TOAST criteria, and relevant arterial steno-occlusive disease (LASO), which was categorized as no stenosis, mild stenosis (<50%), moderate-to-severe stenosis ≥50%, and total occlusion; (5) laboratory data, including white blood cell counts, creatinine serum levels, glucose at presentation, platelet counts, fasting low-density lipoprotein (LDL) cholesterol, and systolic blood pressure; and (5) in-hospital and discharge treatments, including antihypertensives, statins, lipid-lowering treatments other than statins, and antidiabetic therapies.

*Definitions of outcome events*

| Outcome Event | Definition | Operational definition |
| --- | --- | --- |
| Recurrent stroke  (<3 weeks) | Development of early neurological deterioration associated with new lesions documented by relevant neuroimaging study | Discrete new lesions documented by DWI or CT.  If discrete, new lesions within the vascular territory of the index stroke lesion may be counted.  Do not count for increased volume of the index stroke lesions.  Do not count for edema, mass effect, herniation, or hemorrhagic transformation of the index stroke lesions. |
| Recurrent stroke  (≥3 weeks) | Rapidly developing clinical signs of focal (or global) disturbance of cerebral function, with symptoms lasting 24 hours or longer or leading to death, with no apparent cause other than of vascular origin | Data collected by face-to-face or telephone interview with the patient or next of kin.  Question: Were you diagnosed with ischemic stroke or hemorrhagic stroke by any doctor after discharge? |
| Myocardial infarction |  | For END events (≤3weeks after index stroke, more than two from below;  • Typical chest pain  • Troponin elevation  • ECG changes (new ST segment changes, new Q wave, or new left bundle branch block)  For long-term outcomes (≥ 3 weeks after index stroke), data collected through face-to-face or telephone interview with the patient or next of kin.  Question: Were you diagnosed with myocardial infarction by any doctors after discharge? |
| All-cause mortality | Death | Data collected by face-to-face or telephone interview with the patient or next of kin. |

Supplemental Table 1. DLC model hyper-parameters

| **Hyper-parameters** | **Values** |
| --- | --- |
| Number of Clusters | 3 |
| Number of epochs | 30 |
| Number of Layers | [1,2,3] |
| Number of Units | [64,128,256,512] |
| Activation function | [Tanh, ReLU] |
| Batch normalization | [True, False] |
| Learning rate | [0.01, 0.001] |
| Batch size | [256,512] |
| Mini batch size | [32,64] |

Supplemental Table 2. Characteristics of subjects according to the clusters by DLC-Kuiper UB methods (Training set).

|  | Cluster 0 | Cluster 1 | Cluster 2 | P-value |
| --- | --- | --- | --- | --- |
| N | 475 | 2,036 | 2,079 |  |
| Age, yr, mean (SD) | 77.89±8.362 | 69.28±11.511 | 65.59±13.179 | <0.001 |
| Male, n (%) | 192(40.4%) | 1,066(52.4%) | 1,399(67.3%) | <0.001 |
| Arrival time |  |  |  | <0.001 |
| Within 24 h | 427(89.9%) | 1,494(73.4%) | 1,594(76.7%) |  |
| Beyond 24 h | 48(10.1%) | 542(26.6%) | 485(23.3%) |  |
| BMI, mean (SD) | 22.24±3.548 | 23.95±3.278 | 23.40±3.129 | <0.001 |
| Initial NIHSS, med (IQR) | 13.92±5.168 | 5.62±5.209 | 3.56±3.957 | <0.001 |
| Prestroke mRS >1), n (%) | 149(31.4%) | 511(25.1%) | 35(1.7%) | <0.001 |
| TOAST classification |  |  |  | <0.001 |
| LAA | 64(13.5%) | 661(32.5%) | 588(28.3%) |  |
| SVO | 1(0.2%) | 15(0.7%) | 475(22.8%) |  |
| CE | 304(64.0%) | 641(31.5%) | 205(9.9%) |  |
| OE | 6(1.3%) | 43(2.1%) | 35(1.7%) |  |
| UD | 100(21.1%) | 676(33.2%) | 776(37.3%) |  |
| Medical history |  |  |  |  |
| History of TIA | 5(1.1%) | 39(1.9%) | 30(1.4%) | 0.287 |
| History of stroke | 121(25.5%) | 413(20.3%) | 236(11.4%) | <0.001 |
| History of PAD | 13(2.7%) | 4(0.2%) | 11(0.5%) | <0.001 |
| History of CAD | 35(7.4%) | 174(8.5%) | 49(2.4%) | <0.001 |
| HTN | 297(62.5%) | 1,761(86.5%) | 608(29.2%) | <0.001 |
| DM | 136(28.6%) | 741(36.4%) | 332(16.0%) | <0.001 |
| Dyslipidemia | 37(7.8%) | 354(17.4%) | 316(15.2%) | <0.001 |
| Smoking |  |  |  | <0.001 |
| Never | 314(66.1%) | 1,631(80.1%) | 1,144(55.0%) |  |
| Current | 74(15.6%) | 266(13.1%) | 556(26.7%) |  |
| Ex-smoker (quit ≥5 yrs.) | 44(9.3%) | 41(2.0%) | 167(8.0%) |  |
| Recent-smoker (quit <5 yrs.) | 43(9.1%) | 98(4.8%) | 212(10.2%) |  |
| Atrial fibrillation | 311(65.5%) | 669(32.9%) | 186(8.9%) | <0.001 |
| High risk of cardioembolism | 281(59.2%) | 632(31.0%) | 143(6.9%) | <0.001 |
| Congestive heart failure | 14(2.9%) | 1(0.0%) | 3(0.1%) | <0.001 |
| Medication history |  |  |  |  |
| Antiplatelet | 164(34.5%) | 575(28.2%) | 302(14.5%) | <0.001 |
| Anticoagulant | 4(0.8%) | 172(8.4%) | 26(1.3%) | <0.001 |
| Anti-hypertensive | 263(55.4%) | 1,430(70.2%) | 490(23.6%) | <0.001 |
| Anti-diabetics | 109(22.9%) | 644(31.6%) | 246(11.8%) | <0.001 |
| Statin | 53(11.2%) | 329(16.2%) | 127(6.1%) | <0.001 |
| Laboratory findings, mean (SD) |  |  |  |  |
| White blood cell count, 10^3^/µL | 9.70±3.867 | 8.43±2.935 | 8.11±2.744 | <0.001 |
| Hemoglobin, mg/dL | 1.14±1.259 | 0.89±0.604 | 0.84±0.592 | <0.001 |
| Platelet counts , 10^3^/µL | 12.75±2.077 | 13.48±1.871 | 13.83±1.746 | <0.001 |
| Glucose, mg/dL | 215.86±75.362 | 221.18±66.731 | 222.29±59.461 | 0.147 |
| Creatinine, mg/dL | 147.67±60.528 | 139.98±58.033 | 130.28±48.430 | <0.001 |
| Systolic blood pressure, mmHg | 139.76±25.303 | 137.61±23.023 | 139.71±22.589 | 0.009 |
| Large artery disease |  |  |  | <0.001 |
| No stenosis | 75(15.8%) | 539(26.5%) | 1,277(61.4%) |  |
| Mild stenosis <50% | 3(0.6%) | 185(9.1%) | 94(4.5%) |  |
| Significant stenosis >50% | 27(5.7%) | 370(18.2%) | 276(13.3%) |  |
| Complete occlusion | 370(77.9%) | 942(46.3%) | 432(20.8%) |  |

Supplemental Table 3. One-year outcomes of patient groups according to the cluster method (K=3/Training set).

|  | Cluster 0 | Cluster 1 | Cluster 2 | P-value |
| --- | --- | --- | --- | --- |
| (A) K-prototype |  |  |  |  |
| N | 1361 | 468 | 2761 |  |
| Primary outcome, n (event rates) | 159(11.7%) | 70(15.0%) | 374(13.5%) | 0.117 |
| Stroke | 2(0.1%) | 0(0.0%) | 5(0.2%) | 0.649 |
| MI | 120(8.8%) | 53(11.3%) | 314(11.4%) | 0.038 |
| All-cause mortality | 42(3.1%) | 18(3.8%) | 70(2.5%) | 0.229 |
| (B) SSC-Bair |  |  |  |  |
| N | 2,764 | 463 | 1,363 |  |
| Primary outcome | 375(13.6%) | 70(15.1%) | 158(11.6%) | 0.087 |
| Stroke | 70(2.5%) | 18(3.9%) | 42(3.1%) | 0.214 |
| MI | 5(0.2%) | 0(0.0%) | 2(0.1%) | 0.652 |
| All-cause mortality | 315(11.4%) | 53(11.4%) | 119(8.7%) | 0.027 |
| (C) DLC-MMD |  |  |  |  |
| N | 2,973 | 1,517 | 100 |  |
| Primary outcome | 190(6.4%) | 349(23.0%) | 64(64.0%) | <0.001 |
| Stroke | 76(2.6%) | 43(2.8%) | 11(11.0%) | <0.001 |
| MI | 5(0.2%) | 2(0.1%) | 0(0.0%) | 0.885 |
| All-cause mortality | 118(4.0%) | 312(20.6%) | 57(57.0%) | <0.001 |
| (D) DLC-Kuiper |  |  |  |  |
| N | 475 | 2,036 | 2,079 |  |
| Primary outcome | 255(53.7%) | 218(10.7%) | 130(6.3%) | <0.001 |
| Stroke | 20(4.2%) | 67(3.3%) | 43(2.1%) | 0.01 |
| MI | 0(0.0%) | 2(0.1%) | 5(0.2%) | 0.337 |
| All-cause mortality | 240(50.5%) | 155(7.6%) | 92(4.4%) | <0.001 |

P-value; log-rank test

Supplemental Table 4. One-year outcomes of patient groups according to the cluster method (K=3/Validation set).

|  | Cluster 0 | Cluster 1 | Cluster 2 | P-value |
| --- | --- | --- | --- | --- |
| (A) K-prototype |  |  |  |  |
| N | 975 | 175 | 380 |  |
| Primary outcome | 118(12.1%) | 33(18.9%) | 50(13.2%) | 0.052 |
| Stroke | 30(3.1%) | 7(4.0%) | 9(2.4%) | 0.566 |
| MI | 0(0.0%) | 2(1.1%) | 2(0.5%) | 0.012 |
| All-cause mortality | 98(10.1%) | 25(14.3%) | 42(11.1%) | 0.246 |
| (B) SSC-Bair |  |  |  |  |
| N | 175 | 979 | 376 |  |
| Primary outcome | 33(18.9%) | 118(12.1%) | 50(13.3%) | 0.049 |
| Stroke | 7(4.0%) | 30(3.1%) | 9(2.4%) | 0.58 |
| MI | 2(1.1%) | 0(0.0%) | 2(0.5%) | 0.012 |
| All-cause mortality | 25(14.3%) | 98(10.0%) | 42(11.2%) | 0.235 |
| (C) DLC-MMD |  |  |  |  |
| N | 994 | 497 | 39 |  |
| Primary outcome | 64(6.4%) | 123(24.7%) | 14(35.9%) | <0.001 |
| Stroke | 23(2.3%) | 22(4.4%) | 1(2.6%) | 0.078 |
| MI | 2(0.2%) | 1(0.2%) | 1(2.6%) | 0.017 |
| All-cause mortality | 43(4.3%) | 109(21.9%) | 13(33.3%) | <0.001 |
| (D) DLC-Kuiper |  |  |  |  |
| N | 164 | 669 | 697 |  |
| Primary outcome | 63(38.4%) | 88(13.2%) | 50(7.2%) | <0.001 |
| Stroke | 5(3.0%) | 28(4.2%) | 13(1.9%) | 0.043 |
| MI | 1(0.6%) | 2(0.3%) | 1(0.1%) | 0.557 |
| All-cause mortality | 61(37.2%) | 67(10.0%) | 37(5.3%) | <0.001 |

P-value; log-rank test

Supplemental Table 5. One-year outcomes of patient groups according to the SPI-II method.

|  | Low risk | Intermediate risk | High risk | P-value |
| --- | --- | --- | --- | --- |
| (A) All patients |  |  |  |  |
| N | 2,317 | 3,840 | 1,493 |  |
| Primary outcome | 130(5.6%) | 566(14.7%) | 309(20.7%) | <0.001 |
| Stroke | 43(1.9%) | 120(3.1%) | 56(3.8%) | 0.001 |
| MI | 1(0.0%) | 6(0.2%) | 6(0.4%) | 0.031 |
| All-cause mortality | 94(4.1%) | 467(12.2%) | 259(17.3%) | <0.001 |
| (B) training set |  |  |  |  |
| N | 1,407 | 2,311 | 872 |  |
| Primary outcome | 78(5.5%) | 343(14.8%) | 182(20.9%) | <0.001 |
| Stroke | 28(2.0%) | 63(2.7%) | 39(4.5%) | 0.002 |
| Mi | 1(0.1%) | 4(0.2%) | 2(0.2%) | 0.602 |
| All-cause mortality | 55(3.9%) | 286(12.4%) | 146(16.7%) | <0.001 |
| (C) validation set |  |  |  |  |
| N | 443 | 773 | 314 |  |
| Primary outcome | 21(4.7%) | 107(13.8%) | 73(23.2%) | <0.001 |
| Stroke | 5(1.1%) | 31(4.0%) | 10(3.2%) | 0.018 |
| Mi | 0(0.0%) | 1(0.1%) | 3(1.0%) | 0.024 |
| All-cause mortality | 17(3.8%) | 85(11.0%) | 63(20.1%) | <0.001 |
| (D) Test set |  |  |  |  |
| N | 467 | 756 | 307 |  |
| Primary outcome | 31(6.6%) | 116(15.3%) | 54(17.6%) | <0.001 |
| Stroke | 10(2.1%) | 26(3.4%) | 7(2.3%) | 0.337 |
| Mi | 0(0.0%) | 1(0.1%) | 1(0.3%) | 0.471 |
| All-cause mortality | 22(4.7%) | 96(12.7%) | 50(16.3%) | <0.001 |

P-value; log-rank test

Supplemental Figure 1. Selection of the study population

56,853 patients with acute stroke in the CRCS-K registry (Jan 2011- November 2018)

48,717 patients excluded because of admission to

other hospitals

8,136 patients admitted in the CNUH

486 patients excluded

No ischemic stroke (TIA or hemorrhagic stroke) (n=291)

Admission beyond 7 days of onset (n=100)

Lost to follow-up or no information of last follow-up (n=95)

7,650 patients included in the analysis
